# The effect of concomitant chronic kidney disease on the recurrence of atrial fibrillation after catheter ablation: A protocol for systematic review and meta-analysis

**DOI:** 10.1097/MD.0000000000025903

**Published:** 2021-05-21

**Authors:** Xiaodong Liu, Dapeng Wang, Bo Tang, Xiuying Lv

**Affiliations:** a Department of Cardiology, Rizhao Hospital of Traditional Chinese Medicine, Shandong; b Department of Emergency, Suzhou Municipal Hospital, Anhui, China.

**Keywords:** atrial fibrillation, catheter ablation, chronic kidney disease, meta-analysis, protocol

## Abstract

**Background:**

Some new trials have reported the effectiveness of chronic kidney disease on recurrence of atrial fibrillation following catheter ablation. Limited by small number of studies and insufficient outcomes, previous meta-analyses also failed to draw a consistent conclusion on this topic. We thus conducted a new meta-analysis to systematically analyze the effect of chronic kidney disease on recurrence of atrial fibrillation following catheter ablation.

**Methods:**

Two independent investigators followed The Preferred Reporting Items for Systematic Reviews and Meta-Analyses (PRISMA) reporting guidelines to conduct the present meta-analysis. From the inception to June 2021, the EMBASE, PubMed, Web of Science, and Cochrane Library electronic databases were searched using the key phrases “atrial fibrillation,” “chronic kidney disease,” “catheter ablation,” “renal failure,” “renal function,” “renal insufficiency,” “end-stage renal disease,” and “dialysis” for all relevant English-language trials. Observational or randomized controlled trial focusing on assessing the effectiveness of chronic kidney disease on recurrence of atrial fibrillation following catheter ablation was included. *P* < .05 was set as the significance level.

**Results:**

Our hypothesis was that chronic kidney disease is associated with increased atrial fibrosis and a higher risk of arrhythmia recurrence and that restoration of normal rhythm through catheter ablation is associated with improved kidney function.

**Registration number:**

10.17605/OSF.IO/3WJAE.

## 1 Introduction

Chronic renal disease and atrial fibrillation are common clinical comorbid conditions with a complex relationship. In addition to common risk factors such as advanced age, diabetes, and hypertension, the presence of chronic kidney disease independently increases the risk of atrial fibrillation.^[1–3]^ Patients with both conditions also have an increased risk of stroke and anticoagulation-related hemorrhage.^[4,5]^ In addition, atrial fibrillation may accelerate the deterioration of renal function, which can be maintained or improved by maintaining sinus rhythm.^[6]^

Catheter ablation of atrial fibrillation has been a successful treatment option for patients with paroxysmal and persistent atrial fibrillation.^[7]^ Despite technical improvements in ablation and the long-term success of ablation, recurrence of atrial fibrillation after ablation remains a major problem.^[8,9]^ Several parameters, such as persistent atrial fibrillation, left atrial volume, inflammation, and concomitant metabolic syndrome, have been reported as predictors of atrial fibrillation recurrence after radiofrequency ablation.^[10,11]^

To our knowledge, some new trials have reported the effectiveness of chronic kidney disease on recurrence of atrial fibrillation following catheter ablation.^[12–14]^ Limited by small number of studies and insufficient outcomes, previous meta-analyses also failed to draw a consistent conclusion on this topic.^[15,16]^ We thus conducted a new meta-analysis to systematically analyze the effect of chronic kidney disease on recurrence of atrial fibrillation following catheter ablation. Our hypothesis was that chronic kidney disease is associated with increased atrial fibrosis and a higher risk of arrhythmia recurrence and that restoration of normal rhythm through catheter ablation is associated with improved kidney function.

## 2 Materials and methods

### 2.1 Search strategy

Two independent investigators followed The Preferred Reporting Items for Systematic Reviews and Meta-Analyses (PRISMA) reporting guidelines to conduct the present meta-analysis. From the inception to June 2021, the EMBASE, PubMed, Web of Science, and Cochrane Library electronic databases were searched using the key phrases “atrial fibrillation,” “chronic kidney disease,”“catheter ablation,” “renal failure,” “renal function,” “renal insufficiency,” “end-stage renal disease,” and “dialysis” for all relevant English-language trials. Moreover, references cited by the relevant sources were also hand-searched to identify any additional articles that could not be found in our database query. Ethical approval and patient consent were not required because this study was conducted based on previous studies. The systematic review protocol has been registered on Open Science Framework registries with registration number 10.17605/OSF.IO/3WJAE.

### 2.2 Eligibility criteria

Study included in our meta-analysis had to meet the following criteria: observational or randomized controlled trial (RCT) focusing on assessing the effectiveness of chronic kidney disease on recurrence of atrial fibrillation following catheter ablation; the following outcome measures was reported: recurrence of atrial fibrillation, occurrence of thromboembolic events, and 90-day readmissions. Duplicate reports and conference abstracts were excluded. Case reports, biochemical trials, letters, and reviews were also eliminated. Two independent authors screen the titles and abstracts of potentially relevant studies to determine their eligibility based on the criteria.

### 2.3 Data extraction

Two review authors independently extracted data from the included trials using a preconstructed data extraction form. Disagreements were resolved through discussion with a third review author. The following data were extracted: first author, publication year, study design, sample size, mean age, gender, follow-up period, outcome measures, and comorbidities. Discrepancies in data extracted by the 2 review authors were resolved by consensus. The primary outcome was recurrence of atrial fibrillation after catheter ablation in patients with chronic kidney disease or on dialysis. The secondary outcome included 90-day readmissions and occurrence of thromboembolic events. If insufficient data were reported in the published article or supplementary material provided, the corresponding author was contacted to request further data. When it was not possible to retrieve data, results were not included in the meta-analysis and were reported descriptively.

### 2.4 Statistical analysis

The present study was performed by Review Manager Software (RevMan Version 5.3, The Cochrane Collaboration, Copenhagen, Denmark). We used the Mantel-Haenzel method to calculate the pooled odds ratio. Odds ratio with a 95% confidence interval was assessed for dichotomous outcomes. *P* < .05 was set as the significance level. The heterogeneity was assessed by using the Q test and *I*^2^ statistic. When *I*^2^ ≥ 40%, it was considered to represent significant heterogeneity. All outcomes were pooled on random-effect model. The *Z* test was used to assess the overall effect.

### 2.5 Assessment of methodological quality

The Cochrane risk of bias tool was independently used to evaluate the risk of bias of included RCTs by 2 reviewers. The quality of RCTs was assessed by using following 7 items: random sequence generation, allocation concealment, blinding of participants and personnel, blinding of outcome assessment, incomplete outcome data, selective reporting, and other bias. A modified version of the Downs and Black tool was adopted to evaluate the quality of nonrandomized cohort studies. The modified version consists of 27 items with a total possible score of 29. A score of ≥ 75% indicates high quality, 60% to 74% indicates moderate quality and ≤60% low quality. Two investigators independently evaluated included studies on the 27 criteria, with any discrepancies resolved by a third independent reviewer. Kappa values were used to measure the degree of agreement between the 2 reviewers and were rated as follows: fair, 0.40 to 0.59; good, 0.60 to 0.74; and excellent, 0.75 or more.

## 3 Discussion

Atrial fibrillation is the most common clinically significant cardiac arrhythmia and affects almost 2.3 million people in the US population.^[7]^ To our knowledge, some new trials have reported the effectiveness of chronic kidney disease on recurrence of atrial fibrillation following catheter ablation.^[12–14]^ Limited by small number of studies and insufficient outcomes, previous meta-analyses also failed to draw a consistent conclusion on this topic.^[15,16]^ We thus conducted a new meta-analysis to systematically analyze the effect of chronic kidney disease on recurrence of atrial fibrillation following catheter ablation. Our hypothesis was that chronic kidney disease is associated with increased atrial fibrosis and a higher risk of arrhythmia recurrence and that restoration of normal rhythm through catheter ablation is associated with improved kidney function.

## Author contributions

**Conceptualization:** Xiuying Lv.

**Data curation:** Xiaodong Liu, Dapeng Wang.

**Formal analysis:** Xiaodong Liu, Dapeng Wang.

**Funding acquisition:** Dapeng Wang.

**Investigation:** Xiaodong Liu, Dapeng Wang.

**Methodology:** Xiuying Lv.

**Resources:** Dapeng Wang.

**Software:** Xiaodong Liu.

**Supervision:** Dapeng Wang.

**Validation:** Dapeng Wang.

**Visualization:** Bo Tang.

**Writing – original draft:** Xiaodong Liu.

**Writing – review & editing:** Bo Tang.
